# Penetrating cardiac injury with no initial radiological findings and negative E-FAST: The critical role of clinical judgment

**DOI:** 10.1016/j.tcr.2026.101425

**Published:** 2026-08-07

**Authors:** Alga Kajama, Ana Correia, Renato Chimbalanha, Adalberto Dos Santos

**Affiliations:** a Faculty of Medicine, Agostinho Neto University, Avenida Hoji ya Henda, CP 116, Luanda, Angola; b General surgery department at Josina Machel Hospital, Largo Josina Machel, Luanda, Angola; c University of Beira Interior, Avenida Infante D. Henrique, 6200-506, Covilhã, Portugal

**Keywords:** Penetrating cardiac injury, Thoracic trauma, Exploratory thoracotomy, Haemopericardium, FAST limitations

## Abstract

We report the case of a man in his forties admitted to hospital after being stabbed in the precordial region. The initial chest X-ray and FAST examination revealed nothing abnormal. Despite apparent haemodynamic stability, the patient developed progressive dyspnoea, diaphoresis and signs of circulatory compromise. Due to clinical deterioration, an emergency exploratory thoracotomy was performed. Intraoperative findings revealed haemothorax, haemopericardium, and a perforation of the right atrium associated with pericardial rupture. The cardiac injury was successfully repaired, and the patient recovered favourably. This case demonstrates that initial negative imaging does not rule out significant cardiac injuries in penetrating chest trauma. Careful clinical reassessment and timely surgical intervention remain critical for patient survival.

## Highlights

• Penetrating cardiac injuries may present with initially negative imaging findings.
• E-FAST can be falsely negative in cases of small or decompressing haemopericardium.
• Clinical deterioration should override reassuring initial radiologic studies.
• Early surgical exploration can be life-saving in occult cardiac trauma.

## Introduction

Penetrating cardiac injuries are rare in hospital settings due to their high pre-hospital mortality. Among patients who reach medical care, some may initially present haemodynamically stable and without overt clinical signs of cardiac injury. In such cases, diagnosis may be challenging, especially when early imaging studies are inconclusive [1], [2], [3].

Although focused assessment with sonography in trauma (FAST) and chest radiography are cornerstones of the initial evaluation, their sensitivity may be limited in early or small-volume haemopericardium [4], [5], [6]. Clinical deterioration may therefore be the most reliable indicator of occult cardiac injury [7].

In injuries caused by a bladed weapon, the characteristics of the weapon and the angle of attack can provide important information about the anatomical structures potentially involved [8].

Haemodynamic deterioration or a loss of vital signs in patients with penetrating chest trauma should be regarded as indicative of potentially fatal cardiac injury until proven otherwise [9]. Where traumatic cardiac tamponade is suspected, thoracotomy is the procedure of choice, allowing for pericardiotomy, evacuation of haemopericardium, control of cardiac haemorrhage and restoration of circulatory function [10], [11].

We report a case of penetrating precordial trauma with initially negative imaging and E-FAST, in which evolving clinical findings guided life-saving surgical intervention.

## Case presentation

A male patient in his forties was admitted to the General Surgery Department of a tertiary referral hospital in Luanda after being transferred from a private clinic, where he had been treated for a stab wound to the chest.

At the private institution, the patient was haemodynamically stabilised, the wound was sutured, and a posteroanterior chest X-ray revealed no significant abnormalities. Approximately 4 h later, he was transferred for further evaluation.

Upon arrival at our emergency department, the patient was alert and oriented. His vital signs were within acceptable limits. Physical examination revealed a penetrating stab wound, approximately 2 cm in length, located in the precordial region of the left hemithorax, at the level of the second intercostal space, with a downward trajectory from left to right. The patient complained of severe chest pain, dyspnoea, diaphoresis, and intolerance to the supine position. Pulmonary auscultation demonstrated preserved bilateral vesicular breath sounds without adventitious sounds.

Analgesic therapy was initiated, and the patient remained under close observation.

Approximately 8 h after admission, the patient developed clinical deterioration characterised by diaphoresis, worsening dyspnoea, intolerance to the supine position, cold extremities, weak pulse, and hypophonic heart sounds. Breath sounds were diminished at the base of the left hemithorax.

An extended FAST examination was performed, which did not reveal pericardial effusion. However, repeat chest radiography demonstrated a new left pleural effusion (Fig. 1).

**Fig. 1:**
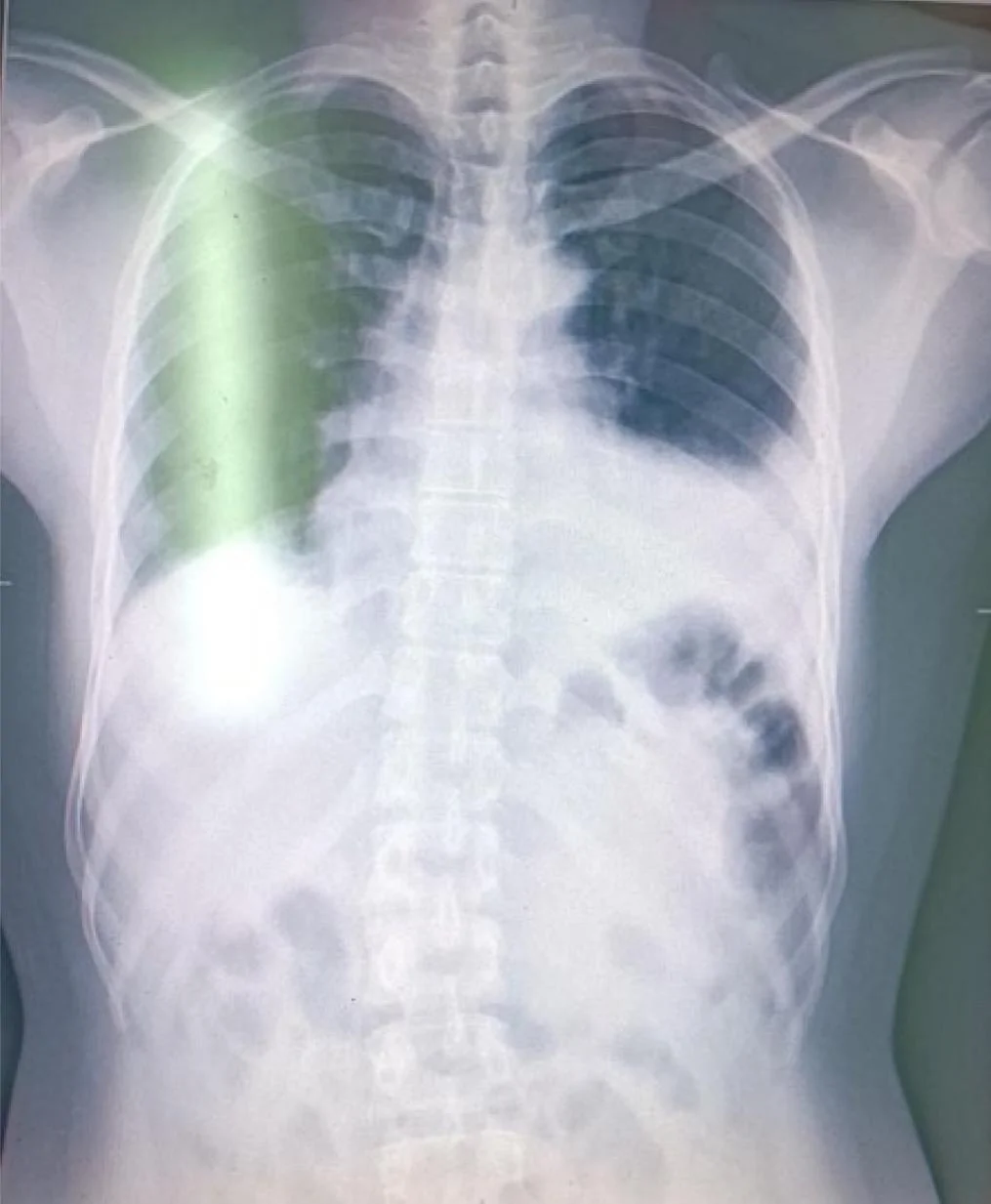
Chest X-ray showing left pleural effusion. Basal opacification of the left hemithorax with obliteration of the costophrenic angle is observed, suggestive of fluid accumulation in the pleural space.

Echocardiographic assessment was not available at the time of clinical deterioration.

Given the persistent symptoms and clinical suspicion of occult cardiac injury, despite negative ultrasound results, an urgent exploratory thoracotomy was performed.

## Surgical procedure and intraoperative findings

A left anterolateral thoracotomy was performed via the appropriate intercostal space. Following access to the pleural cavity, approximately 300 mL of haemothorax was drained. Surgical exploration revealed a rupture of the parietal pleura and pericardium approximately 1 cm in length, located in the upper right portion of the pericardial sac, overlying the right atrium.

Pericardiotomy allowed the drainage of approximately 200 mL of haemopericardium, as well as the removal of clots present in the pericardial cavity. Active bleeding was observed through the pericardial laceration (Fig. 2-B), synchronised with cardiac movements and more evident during diastole, raising strong suspicion of an underlying cardiac lesion. Careful enlargement of the pericardial opening revealed a punctate lesion of the right atrium (Fig. 2-A), responsible for the bleeding.

**Fig. 2:**
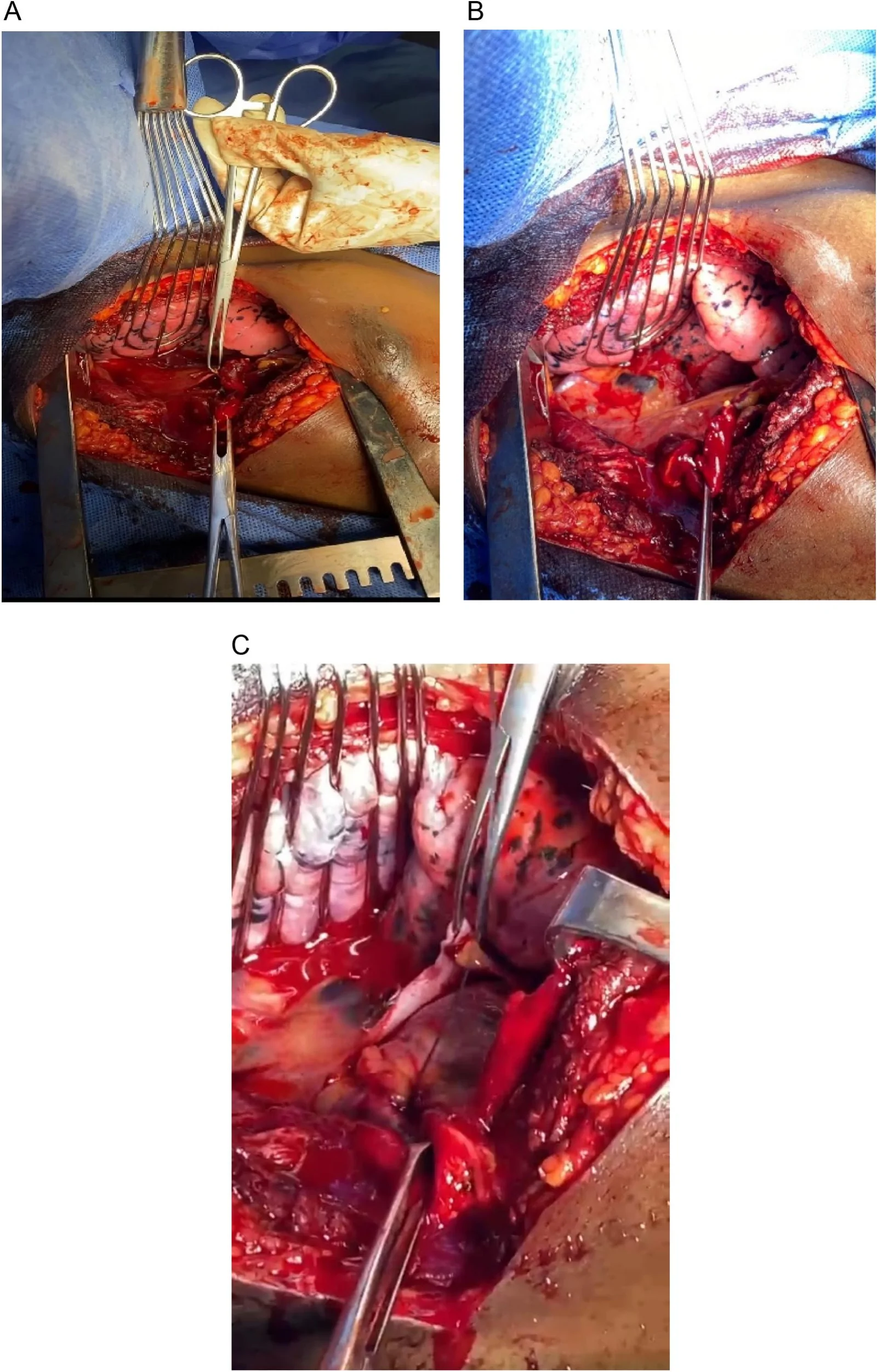
A. Intraoperative view during exploratory thoracotomy. The surgical field shows direct exposure of the heart with active bleeding from a penetrating cardiac injury, requiring immediate surgical control. B. Clots identified in the pericardial window. Intraoperative view showing clotted blood within the pericardial cavity following pericardial window opening, consistent with haemopericardium. C. Intraoperative control of the cardiac injury. The image shows surgical control of the cardiac lesion following pericardial opening and clot evacuation during exploratory thoracotomy.

The atrial lesion was promptly controlled and surgically repaired with a U-shaped suture using Vicryl® 2-0 (Fig. 2-C), achieving complete haemostasis. After confirming the absence of residual bleeding, the pericardium was partially reapproximated with three separate sutures, maintaining an opening for drainage and reducing the risk of cardiac tamponade in the event of suture dehiscence or rebleeding. A water-seal chest drain was subsequently placed prior to closure of the thoracotomy.

## Postoperative course

The patient initially demonstrated favourable recovery. On the fifth postoperative day, he developed mild chest pain and dyspnoea. Physical examination revealed decreased breath sounds in the left hemithorax.

Repeat chest radiography showed moderate left pleural effusion with obliteration of the costophrenic angle (Fig. 3-A). A pleurostomy was performed, draining approximately 600 mL of blood.

**Fig. 3:**
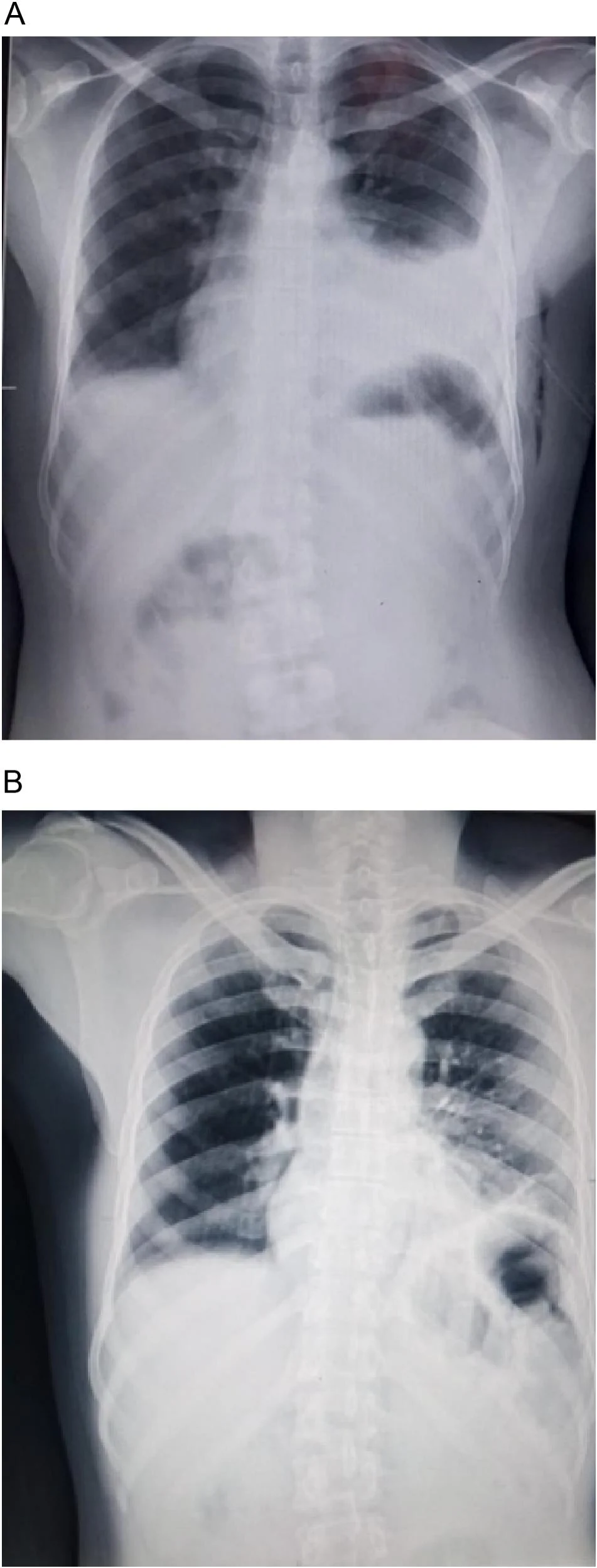
A. Chest X-ray demonstrating left pleural effusion. Chest radiograph showing opacification of the left hemithorax with blunting of the left costophrenic angle, consistent with pleural fluid accumulation after surgery. B. Follow-up chest X-ray demonstrating resolution of the left pleural effusion. The radiograph shows restoration of the left costophrenic angle and re-expansion of the lung following treatment.

Subsequent radiographic evaluation demonstrated significant improvement (Fig. 3-B). Antibiotic therapy with amoxicillin-clavulanate was instituted. The patient was discharged in stable condition and referred for outpatient follow-up.

## Discussion

Penetrating cardiac injuries account for a small proportion of cases of chest trauma, but are associated with extremely high mortality rates. Survival depends largely on rapid diagnosis and immediate surgical intervention [1], [12].

Cardiac tamponade is a common complication; however, in some cases, pericardial rupture allows decompression into the pleural cavity, masking the classic signs and making diagnosis more difficult. This mechanism likely contributed to the initially negative E-FAST scan results in our patient [1].

Although the FAST scan is highly valuable in trauma settings, its sensitivity may be reduced in cases of small or transient haemopericardium, operator dependency, or associated pericardial rupture. Therefore, a negative ultrasound scan does not rule out significant cardiac injury in penetrating precordial trauma.

In this case, clinical deterioration was the decisive factor guiding the decision for surgical exploration.

Although surgical intervention is typically indicated in haemodynamically unstable patients, this case demonstrates that evolving clinical signs in initially stable individuals should lead to aggressive diagnostic and therapeutic consideration.

Early thoracotomy enabled definitive diagnosis and repair of a right atrial injury, preventing potentially fatal complications.

The choice of a left anterolateral thoracotomy was motivated by the patient's clinical deterioration, the presence of a left-sided haemothorax, and the need for immediate thoracic exploration. In penetrating chest trauma, the source of bleeding may remain unclear despite imaging studies, particularly when cardiac injury is not initially evident. In such situations, emergency thoracotomy provides rapid access to the pleural cavity, allowing both diagnosis and haemorrhage control [13], [14]. Although median sternotomy is generally considered the preferred approach for confirmed anterior cardiac injuries in stable patients, the urgency of the clinical situation and local resource limitations favoured a left anterolateral thoracotomy in our case.

This approach is in line with the principles of trauma surgery, which prioritise rapid access and haemorrhage control in unstable patients with suspected intrathoracic haemorrhage, when the exact source of the injury remains uncertain [8], [14].

On the other hand, the small amount of residual pleural effusion observed postoperatively can be explained by the pre-existing left haemothorax and the absence of continuous suction drainage, which may have limited the complete evacuation of residual blood, despite the insertion of a large-bore chest tube.

Although echocardiography is an important diagnostic tool in cases of suspected penetrating cardiac injuries, it was not available in our setting at the time of the patient's clinical deterioration. This limitation highlights the challenges faced in resource-limited settings and reinforces the importance of serial clinical assessment and repeated bedside imaging. In the present case, despite an initially negative *E*-FAST scan, progressive haemodynamic and respiratory deterioration led to surgical exploration, which ultimately confirmed a lesion in the right atrium. This case underscores that clinical judgment must prevail when the level of suspicion remains high, even in the absence of confirmatory imaging studies.

## Conclusion

Penetrating precordial trauma requires continuous clinical reassessment, even when initial imaging studies are negative.

This case underscores that clinical judgment must prevail over initially reassuring radiologic findings. Early surgical exploration, guided by evolving clinical signs, can be life-saving in occult penetrating cardiac injury.

## CRediT authorship contribution statement

**Alga Kajama:** Participated in the surgery, Clinical data acquisition, Conceptualization, Writing – original draft. **Ana Correia:** Surgical management, Data interpretation. **Renato Chimbalanha:** Surgical management, Critical revision of the manuscript. **Adalberto Dos Santos:** Writing – review & editing.

## Ethical statement

Written informed consent was obtained from the patient for publication of this case report and accompanying images.

## Funding sources

The authors received no external funding for this study.

All authors approved the final manuscript.

## Declaration of competing interest

The authors declare that they have no known competing financial interests or personal relationships that could have appeared to influence the work reported in this paper.
